# Supplementary material for: Chromothripsis during telomere crisis is independent of NHEJ, and consistent with a replicative origin
Source: Genome Res. 2019 May;29(5):737–49. doi: 10.1101/gr.240705.118 (PMC6499312; doi:10.1101/gr.240705.118)
Supplement: Supplemental Material [file supp_gr.240705.118_Supplemental_file_1.zip › contigs/annotated_contigs/DB57/contig.2.DB57_length_522_mean_cov_14.7126436782.docx]

**DB57_length_522_mean_cov_14.7126436782**

```
CACACACACACAAATTAGCCATGTGTGGTGGTGGGCGCCTGTAATCCCAGGTACTTGGGAAGCTGAGGCATGAGAATCACTTGAACCTG
 >chr11:45638006-45638451 - E=1e-251
GGAGGCAGAGATTGCAGTGAGCCAAGATTATGCCATTACACTCTAGCCTGGGTGACAGAATGAGACTGTCTCAGAAAAAAAACAACATA

TATACATACATATAATATATATTTAATTTGTATAAATTTATGAGATGCAAGTACAGTTTTGATACATGGATATATTGCATAGTGGTGAA

GTCTGAGCTTTTTGTGTAACCATCACCCAAATCATGTACATTGCACTCCTTAAGTAATTTCTCAGCCCTCACCCCCACATTCTCCCACC

CTCCCTAGTCTCCAATGTCTATTATTCTGCACTCTTGGAATTAGAATTGGATCCGGCTCTTGGAGGTGCTCCTATCTAGTGGGGT|AGA
                                                                                      >c
|GAGAGAGAGAGAGAGACAGACAGACAGAGAGAACGAAAGAGACAGACAGAGAGAGAGAGATGGACAGAGAGAGACAGThrUn_gl000220:127248-127331 - E=3e-32
```
